# Aerosolized amikacin for treatment of pulmonary *Mycobacterium avium *infections: an observational case series

**DOI:** 10.1186/1471-2466-7-2

**Published:** 2007-02-23

**Authors:** Kala K Davis, Peter N Kao, Susan S Jacobs, Stephen J Ruoss

**Affiliations:** 1 Division of Pulmonary and Critical Care Medicine, Stanford University Medical Center, Stanford, CA, USA

## Abstract

**Background:**

Current systemic therapy for nontuberculous mycobacterial pulmonary infection is limited by poor clinical response rates, drug toxicities and side effects. The addition of aerosolized amikacin to standard oral therapy for nontuberculous mycobacterial pulmonary infection may improve treatment efficacy without producing systemic toxicity. This study was undertaken to assess the safety, tolerability and preliminary clinical benefits of the addition of aerosolized amikacin to a standard macrolide-based oral treatment regimen.

**Case Presentations:**

Six HIV-negative patients with *Mycobacterium avium intracellulare *pulmonary infections who had failed standard therapy were administered aerosolized amikacin at 15 mg/kg daily in addition to standard multi-drug macrolide-based oral therapy. Patients were monitored clinically and serial sputum cultures were obtained to assess response to therapy. Symptomatic improvement with radiographic stabilization and eradication of mycobacterium from sputum were considered markers of success.

Of the six patients treated with daily aerosolized amikacin, five responded to therapy. All of the responders achieved symptomatic improvement and four were sputum culture negative after 6 months of therapy. Two patients became re-infected with *Mycobacterium avium intracellulare *after 7 and 21 months of treatment. One of the responders who was initially diagnosed with *Mycobacterium avium intracellulare *became sputum culture positive for *Mycobacterium chelonae *resistant to amikacin after being on intermittent therapy for 4 years. One patient had progressive respiratory failure and died despite additional therapy. There was no evidence of nephrotoxicity or ototoxicity associated with therapy.

**Conclusion:**

Aerosolized delivery of amikacin is a promising adjunct to standard therapy for pulmonary nontuberculous mycobacterial infections. Larger prospective trials are needed to define its optimal role in therapy of this disease.

## Background

Nontuberculous mycobacterial (NTM) pulmonary infection is now recognized as a serious disease in a subset of apparently immunocompetent hosts [1-4]. Though initially described as a disease of Caucasian women, its reported incidence is increasing in other populations such as nonwhites and patients in urban areas, possibly as a result of increased disease awareness and improved clinical investigation efforts. In North America infection rates are approaching 15 per 100,000[5]. *Mycobacterium avium complex *(MAC) has been reported most commonly, followed by rapid grower mycobacterium (RGM) (M. chelonae, M. abscessus and M. fortuitum) and M kansasii [2]. NTM disease manifestation depends on a complex interaction between specific mycobacterial pathogens and the host immune system. Preliminary data has suggested interferon-gamma deficient pathways, presence of a cystic fibrosis gene mutation and alpha-1 antiproteinase gene mutations as possible predisposing factors for pulmonary disease development in immunocompetent patients without underlying lung disease[3,6] Of particular interest is the increasing incidence of MAC lung infections characterized by progressive parenchymal lung disease in middle aged and elderly women with no history of smoking or underlying lung disease[7].

Currently, the accepted standard therapy for patients with MAC infection involves the use of a macrolide-based multi-drug regimen consisting of a macrolide or azalide (clarithromycin or azithromycin), a rifamycin (rifabutin or rifampin) and ethambutol, with or without the initial inclusion of intravenous streptomycin or amikacin. Amikacin (intravenous) and other antibiotics such as fluoroquinolones have largely been reserved for patients with more advanced disease or as salvage therapy[8,9]. Rapid growers such as M. chelonae and M. abscessus are even more difficult to treat and usually require complex medical regimens which include intravenous antibiotics such as cefoxitin, amikacin, imipenem, and linezolid in addition to oral macrolide therapy. The available data on therapy demonstrates that successful eradication of MAC pulmonary infection after 12 months of therapy is achieved in only 50–71% of HIV-negative patients[3,10,11]. Successful therapy has been hampered by potential drug-drug interactions, poor tolerance, toxicity and relapse after completion of treatment [12]. More effective treatment strategies are needed for optimal control of these infections.

Aminoglycosides, particularly amikacin, have been recommended and variably used for limited duration (less than 4 to 6 months) parenteral therapy for NTM infections. The aminoglycosides exhibit significant concentration-dependent bactericidal activity against NTM. More extended parenteral therapy duration has been avoided due to the substantial risks of nephrotoxicity (15%), ototoxicity (37%) and vestibular toxicity (9%)[13]. Aerosol antibiotic delivery offers the potential advantage of achieving high lung drug concentrations with concomitant low systemic absorption, low serum drug concentrations, and thus a diminished risk of systemic toxicities[14]. In a small intensive care unit study[15], aerosolized amikacin delivered to mechanically ventilated patients at doses of 400 mg every 8 hours resulted in mean peak sputum concentrations of more than 5,000 mcg/ml, an impressive level that would be completely unachievable (and toxic) when delivered systemically. Aerosolized antibiotics have been used with notable success in the treatment of chronic infection with *Pseudomonas aeruginosa *in cystic fibrosis[14,16]. Recent studies have expanded on this concept and have demonstrated safety and tolerance of inhaled tobramycin solution for inhalation in the management of non-cystic fibrosis patients with severe bronchiectasis[17]. These studies were able to demonstrate a decrease in the number of hospitalizations, improvement in respiratory symptoms and in health related quality of life[18]. Inhaled aminoglycosides (gentamycin, kanamycin and streptomycin) have also been reported to provide benefit when used as a salvage therapy in complex and refractory pulmonary tuberculosis infections [19,20].

The addition of aerosolized amikacin to standard oral therapy for NTM pulmonary infection has the potential to improve treatment efficacy without increasing the risk for systemic toxicity. Inhaled amikacin has been considered as adjunctive therapy for pulmonary NTM infections for a number of years, and has been used in very limited circumstances, but no formal evaluation of the pharmacokinetics, tolerability or therapeutic efficacy of inhaled amikacin has been undertaken. This current investigation reports the pharmacokinetics of inhaled amikacin in adults, as well as our preliminary experience with inhaled amikacin therapy in a small patient cohort with pulmonary NTM infection.

## Case Presentations

Treatment subjects were identified from the population of patients with pulmonary NTM infections followed and treated at the Stanford University Medical Center Chest Clinic. All patients included in this series had a diagnosis of NTM infection that met the disease diagnostic criteria established in the 1997 ATS consensus statement[9]. These patients had remained sputum culture positive despite a minimum of six months of standard oral therapy with a macrolide-based multi-drug regimen. All of the patients included in this case series were symptomatic and had radiographic evidence of nodular infiltrates, bronchiectasis and/or cavitary disease as outlined in Table 1. Institutional approval for inhaled amikacin delivery was obtained, as was informed consent from all study subjects.

**Table 1:** Case Data Summaries

**Pt. No**	**Sex, Age (Years)**	**NTM Species**	**Radiographic Pattern**	**Duration of therapy prior to starting amikacin (months)**	**Amikacin Therapeutic Regimen**	**Duration of Inhaled Amikacin (months)**	**Current Therapeutic Regimen**	**Current Patient Status**	**Current Sputum Culture Status**
1	F, 73	MAC	nodular infiltrates, bronchiectasis in RUL, RML, lingula	96	CLA, RIF, EMB, inhaled amikacin	9	no oral antibiotics since 7/05; no inhaled amikacin since 6/06.	rare cough, wt loss, sweats, fatigue; abdominal cramps	negative for 21 months; now positive (MAC)
2	F, 67	MAC	bilateral bronchiectasis, cavitary lesion RUL and bilateral apical fibrosis/scarring	12	AZI, RIF, EMB, inhaled amikacin	4	n/a	progressive disease; died	persistent positive at death
3	F, 66	MAC, *M. chelonae*	bronchiectasis RML, RLL, LLL, lingula; bilateral apical fibrosis/scarring, centrilobular nodules	48	AZI, RIF, EMB, inhaled amikacin	52	AZI 500 mg/d since 4/06	status post multiple lobectomies; cough and exertional dyspnea; daily low grade fevers	negative for 6 months., now positive; *M. chelonae *(resistant to amikacin
4	F, 71	MAC	bronchiectasis and centrilobular nodules in posterior segments of both upper lobes, RML, lingula and lower lobes	36	CLA, inhaled amikacin	16	no antibiotics since 11/05	rare cough	negative for 7 months; now positive; MAC (resistant to EMB, RIF)
5	F, 52	MAC, *M. chelonae*	LUL wedge resection for MAC; bronchiectasis in LLL, RUL; R apical scarring, nodules in LLL, LUL, lingula	0.5	AZI, inhaled amikacin (thrombocytopenia on RIF/EMB)	13	inhaled amikacin 1000 mg/d and AZI 250 mg/d since 5/05	improved with some cough, no purulence	negative for 6 mo.
6	F, 54	MAC	bronchiectasis w/bronchial wall thickening RLL, RUL.	13	AZI, inhaled amikacin	8	AZI 500 mg 2/wk, inhaled amikacin 1000 mg 3/wk	rare cough, clinically well	no cough sputum (despite sputum induction)

Abbreviations: AZI (azithromycin); CIP (ciprofloxacin); CLA (clarithromycin); EMB (ethambutol); RIF(rifampin); RUL (right upper lobe); RML (right middle lobe); RLL (right lower lobe); LLL (left lower lobe)

Patients were treated with aerosolized amikacin as a single daily dose of 15 mg/kg administered using a DeVilbiss PulmoAide Compressor/Nebulizer system (DeVilbiss Health Care; Somerset, Pa) in addition to their concurrent oral antibiotic regimens. Amikacin therapy was anticipated to continue for the duration of their oral antibiotic course (estimated 12–18 month course). Patients were pre-treated with an albuterol meter dose inhaler, if they developed shortness of breath or cough with aerosolized amikacin administration.

An inhaled amikacin dose of 15 mg/kg/day was chosen with the following considerations: a) 15 mg/kg is the usual total daily IV dose for therapy with amikacin; b) the goal of inhaled amikacin therapy was to produce lung drug concentrations that significantly exceed minimum inhibitory concentration (MIC), with particular attention to patients with significant anatomic lung problems including bronchiectasis, where lung drug distribution may be much less uniform than in normal lung.

None of the study patients were currently receiving intravenous aminoglycoside therapy. Patients were monitored throughout therapy for evidence of nephrotoxicity, ototoxicity and vestibuar toxicity. Clinical improvement was determined on the basis of a combination of the following: symptomatic improvement, radiographic stabilization or improvement, and eradication of mycobacterium from sputum cultures. Sputum cultures were performed at 8 to 12 week intervals and were induced with hypertonic saline if the patient's cough was non-productive. Aerosolized amikacin was held for 24 hours prior to sputum induction for surveillance cultures.

Six patients with NTM pulmonary infections were treated with aerosolized amikacin at a dose of 15 mg/kg/day in addition to a concurrent standard oral macrolide based multi-drug regimen. Table 1 outlines specific patient characteristics. All of the patients were women between the ages of 52 and 71 years. All had been treated previously with standard oral macrolide/rifamycin-based regimens and either had failed to respond or were unable to tolerate therapy due to side effects. Patient 4 was infected with a MAC species that was resistant to ethambutol and rifampin, necessitating a change in medications. All remained symptomatic despite prior interventions and had progressive or persistent symptoms including fatigue, cough, hemoptysis, dyspnea, and weight loss. None of the patients had known macrolide resistant NTM infections. Each had a different radiographic presentation on chest radiograph and CT. Patient 1 had primarily nodular infiltrates with mild multi-lobar bronchiectasis; patient 2 had severe multi-lobar bronchiectasis and cavitary nodules; patient 3 had multi-lobar bronchiectasis, centrilobular nodules and a history of a right middle lobe and lingual resection; patient 4 had mild multi-lobar bronchiectasis and diffuse centrilobular nodules; patient 5 had multi-lobar bronchiectasis, centrilobular nodules and a history of a left upper lobe wedge resection; patient 6 had focal bronchiectasis and bronchial wall thickening confined to the right upper and lower lobes. Patients received an average of 750 mg to 1000 mg/day of aerosolized amikacin based on weight.

Patients 1, 3, 4, 5 and 6 tolerated therapy well. They all noted an improvement in cough and fatigue with therapy and were sputum culture negative for NTM on follow-up cultures after 6 months of therapy. Patient 3 developed recurrent NTM disease after 6 months of culture-negative sputum. Her initial sputum cultures prior to initiating inhaled amikacin therapy revealed MAC; subsequent cultures have grown *M. chelonae *and *M. gordonae*. Patient 2 was unable to tolerate prolonged therapy with aerosolized amikacin and discontinued therapy after 4 months because of cough and inconvenience of administration. She continued to experience progressive symptoms after cessation of therapy and died of respiratory failure.

Side effects of therapy included voice hoarseness, sore throat (transient) and oral candidiasis. Two patients developed oral candidiasis on aerosolized amikacin therapy that was not responsive to Nystatin oral rinse. Both cases responded well to oral fluconazole therapy. Subsequent episodes of oropharyngeal problems were prevented with improved vigilance of mouth care (including mouth gargling as well as rinsing) after each dose administration. There was no evidence of nephrotoxicity, ototoxicity or vestibular toxicity with therapy.

## Conclusion

The treatment of NTM pulmonary infections has been punctuated by intolerance of therapy, side effects and recurrence of infection. The addition of aerosolized amikacin at 15 mg/kg/day to a standard oral macrolide-based regimen led to successful treatment of NTM pulmonary disease in 66.7% (4/6) of patients treated. Aerosolized amikacin, at the dose used in this case series, is well tolerated, lacks any evidence of systemic toxicity (particularly ototoxicity and nephrotoxicity) and is of potential benefit in patients who have failed or were intolerant of standard macrolide-based therapy. Patients with prior therapeutic failures are notable for their typically poor response rates with subsequent treatment regimens, which make the preliminary responses seen in this study even more significant. Most of our patients demonstrated clinical improvement in symptoms on aerosolized amikacin with diminished cough, sputum production and fatigue. Patients who failed to respond to inhaled amikacin therapy in our case series had more extensive parenchymal involvement and cavitary disease on high resolution CT (HRCT) scans, were sputum culture positive for *M. chelonae*, and/or received inhaled amikacin therapy for less than four months.

Patients with NTM infections and extensive parenchymal involvement are known to have poorer outcomes [3]. Successful clearance of organisms becomes more difficult in the face of a combination of potentially very important factors including altered lung anatomy and function, and patients' inherent increased susceptibility to NTM. As a result, the likelihood of relapse and/or re-infection with other NTM is quite substantial [21,22]. In our case series, patient 3 had MAC and later developed M. chelonae after clearing her initial infection and patient 5 was co-infected with both organisms at the onset of treatment, which also raises the question of differing susceptibilities and response to treatment depending on the organism.

There is an increasing view among investigators, particularly related to inhaled aminoglycoside therapy in cystic fibrosis, that increased delivered drug dose, and the resultant increased lung drug concentrations, might be associated with improved therapeutic utility. In an independent study performed by our group, aerosolized amikacin administered to healthy volunteers at a dose of 5 mg/kg, produced sputum concentrations of 25–298 mcg/ml 1-hour post administration. These sputum concentrations far exceed the MIC_90 _of amikacin for the vast majority of NTM isolates[23], while producing serum amikacin concentrations that are at or below the accepted range for trough concentrations produced by intravenous amikacin administration[13-15,24]. These results are consistent with those published for the use of inhaled tobramycin, where measured serum levels after inhaled drug delivery remain very low, even with doses that exceed usual total daily parenteral doses[16].

This study has a number of limitations. The reported follow-up of patients in our case series occurred over a 9–52 month period. Longer follow-up of each patient may be needed to fully assess disease recurrence (either by re-infection or by reactivation of suppressed initial infection) over time in responders, particularly given the apparent increased inherent susceptibility of these patients to NTM infections. In our study, aerosolized amikacin was administered using a DeVilbiss PulmoAide Compressor/Nebulizer system (DeVilbiss Health Care; Somerset, Pa). We do not know the aerosol droplet size that would be best to generate optimal distribution of amikacin in patients with pulmonary NTM infection. Other aerosol delivery systems may be of equal or greater utility for amikacin delivery in these circumstances.

Aerosolized amikacin is a promising addition to the current therapies used for the treatment of pulmonary NTM infections. It is well tolerated and in our series was able to stabilize or clinically improve patients who had failed standard macrolide-based NTM therapy. We have not addressed a number of important additional aspects of the use of aerosolized amikacin in this disease, including the optimal dose, optimal treatment duration, or the optimal concomitant drug regimen to be used with inhaled amikacin. The question of whether earlier and/or more aggressive treatment for NTM infection in this patient population would lead to decreased morbidity and improved outcomes also cannot be answered by this study. These are, however, extremely important questions that will be best addressed in a larger, randomized, prospective treatment trial.

## Abreviations

NTM: nontuberculous mycobacterial

## Competing interests

The author(s) declare that they have no competing interests.

## Authors' contributions

KD – analysis/interpretation of data, involved in the drafting/revision of manuscript; and has read and given approval of final draft.

SJ – patient data collection, involved in the drafting/revision of manuscript; and has read and has given approval of final draft.

PK – study conception and design, analysis of data, patient management, drafting of manuscript; and has read and has given approval of final draft.

SR – study conception and design, analysis of data, patient management, drafting of manuscript; and has read and has given approval of final draft

## Pre-publication history

The pre-publication history for this paper can be accessed here:


